# Investigating Factors Related to Length of Hospital Stay in Male Patients With Lung Cancer Undergoing Chemotherapy

**DOI:** 10.7759/cureus.87717

**Published:** 2025-07-11

**Authors:** Takeshi Yamazaki, Minami Tanemura, Michiko Tsuchiya, Yukio Nagasaka, Shota Kotani, Shojiro Egoshi, Kohji Iwai, Jun Horie

**Affiliations:** 1 Department of Physical Therapy, Faculty of Health Sciences, Kyoto Tachibana University, Kyoto, JPN; 2 Department of Rehabilitation, Rakuwakai Otowa Hospital, Kyoto, JPN; 3 Department of Respiratory Medicine, Rakuwakai Otowa Hospital, Kyoto, JPN; 4 Rakuwakai Kyoto Respiratory Center, Rakuwakai Otowa Hospital, Kyoto, JPN; 5 Department of Physical Therapy, Faculty of Rehabilitation Science, Kobe International University, Kobe, JPN; 6 Faculty of Health Sciences, Hiroshima Cosmopolitan University, Hiroshima, JPN; 7 Department of Physical Therapy, Faculty of Care and Rehabilitation, Seijoh University, Tokai, JPN

**Keywords:** adverse events, albumin, chemotherapy, length of stay, lung cancer

## Abstract

Background/purpose: In recent years, length of hospital stay has decreased, necessitating smoother discharge support. However, in patients with cancer receiving chemotherapy, longer hospital stays are associated with an increased incidence of adverse events. Moreover, no studies have examined indicators of the risk of prolonged hospitalization before treatment initiation. Specifically, reports clarifying the relationship between blood data, such as serum albumin (albumin) levels, and length of hospital stay are lacking. Therefore, this study aimed to investigate the characteristics of male patients with lung cancer undergoing chemotherapy and examine the factors related to length of hospital stay.

Materials and methods: This was a cohort study of 42 male patients with lung cancer undergoing chemotherapy (mean age: 70 ± 6 years; body mass index: 22 ± 3 kg/m²; Charlson comorbidity index: 4 ± 2 points; performance status (PS): 1 ± 0). Measurement items included comorbidities, frailty, PS, the functional independence measure, the Barthel Index, and biochemical data. Multiple regression analysis was used to examine the influence of each indicator on length of hospital stay, with a significance level of 5%.

Results: Multiple regression analysis showed a significant association between albumin levels at admission and length of hospital stay (β = -0.512, t = -3.624, p < 0.001).

Conclusion: The results of this study suggest that lower albumin levels at admission may lead to prolonged hospital stays in male patients with lung cancer undergoing chemotherapy. These findings indicate that a poor albumin status before chemotherapy may be related to length of hospital stay.

## Introduction

Annually, 370,000 people in Japan die from malignant neoplasms, with lung cancer being the leading cause of cancer-related mortality. The number of new cancer cases has reached 860,000 annually. This indicates that one in two Japanese people will develop cancer, and one in three will die from it [1]. Moreover, lung cancer continues to be the primary cause of cancer-related deaths among men [2].

Remarkable progress has been made in lung cancer treatment over the past 20 years. Various anticancer drugs exist, including traditional cytotoxic agents, molecular-targeted therapies indicated for specific genetic abnormalities, and immune checkpoint inhibitors, which have recently emerged as a third-line treatment. The decision to administer cytotoxic anticancer drugs is based not only on the cancer's histological type and stage but also on various factors, including the patient’s chronological age, organ function, and general health condition. However, older patients with cancer often exhibit significant individual differences in physical and organ function, as well as comorbidities. These factors may increase the risk of severe adverse events when standard treatments are administered. Therefore, in some cases, treatment is provided through hospitalization rather than outpatient-based care pathways [3].

Advancements in medical care, improvements in treatment techniques, and enhancements in home and community-based healthcare have led to shorter hospital stays. Consequently, an increasing number of patients are transitioning to home care or community care services much earlier. The disadvantages of prolonged hospitalization for patients include increased mental and physical burdens, restricted daily activities, and delayed social reintegration. For hospitals, these disadvantages include a shortage of beds and medical resources, as well as an increased workload for healthcare staff. For both patients and hospitals, prolonged hospitalization increases medical costs and increases the risk of hospital-acquired infections. Therefore, early recovery and appropriate discharge are desirable.

In patients with cancer undergoing chemotherapy, advanced age and frailty increase the incidence of chemotherapy-induced adverse events, which can prolong length of hospital stay [4-8].

However, no studies have examined the effect of physical function at the time of hospital admission, before adverse events occur, on length of hospital stay, even in patients with lung cancer during the initial course of treatment, which is typically provided during hospitalization.

Conversely, several studies in other disease populations have reported associations between blood biomarkers and length of hospital stay [9].

Therefore, this study aimed to investigate whether pretreatment indicators, such as serum albumin (albumin) levels, affect length of hospital stay in older male patients with lung cancer receiving their initial inpatient chemotherapy. This study focused on lung cancer, which has the highest mortality rate among men in Japan [2]. Identifying patients requiring preventive interventions from the time of admission will enable more efficient discharge planning. As the number of cancer patients is expected to increase, more effective approaches are anticipated.

## Materials and methods

Participants and covariates

This cohort study included 44 male patients with lung cancer undergoing chemotherapy who were hospitalized for their initial chemotherapy regimen or upon a regimen change at Rakuwakai Otowa Hospital between April 2019 and March 2024. The timing of physical function assessments was standardized to either the time of hospitalization or before the initiation of chemotherapy.

Exclusion criteria included severe comorbid internal diseases other than lung cancer, combination therapy with radiation, treatments other than lung cancer therapy, gait disorders, severe dementia, and cases wherein the attending physician determined that research cooperation could not be obtained.

The sample size was calculated using G*Power version 3.1.9.7 (Heinrich Heine University Düsseldorf, Düsseldorf, Germany). To detect factors associated with length of hospital stay using multiple regression analysis, a sample size of 41 participants was estimated, assuming a significance level of 5%, statistical power of 0.8, effect size of 0.3, and four independent variables.

Muscle strength evaluation

Grip strength in the dominant hand was assessed using a TOEI LIGHT handgrip dynamometer (Takei Scientific Instruments Co., Ltd., Niigata, Japan). Each measurement was performed twice on both sides, and the highest value from each side was recorded and analyzed.

Charlson comorbidity index

The Charlson comorbidity index (CCI) quantifies the severity of comorbidities for evaluation. The main feature is that the score is calculated by summing the weighted scores of 19 comorbidities. The scoring system assigns different weights to each condition as follows: myocardial infarction, congestive heart failure, peripheral vascular disease, cerebrovascular disease, dementia, chronic lung disease, connective tissue disorders, peptic ulcers, mild liver disease, and diabetes are each given one point. Conditions such as hemiplegia, moderate-to-severe kidney disease, diabetes with organ complications, malignant tumors, leukemia, and lymphoma are assigned two points. Moderate-to-severe liver disease is weighted at three points, while metastatic solid cancer and AIDS receive the highest score of six points. This scoring method is commonly utilized to predict prognosis and estimate mortality risk, with higher scores indicating a greater likelihood of a poor outcome [10].

Eastern Cooperative Oncology Group (ECOG) performance status (PS) scale

This is a simple indicator used to assess the overall functional status of a patient. It is primarily used to evaluate the appropriateness of the cancer treatment and prognosis. The PS scale assesses the patient's level of independence in daily activities using a five-point rating system ranging from 0 to 5 (Table 1). This scale is widely used in determining treatment plans and is a criterion for patient selection in clinical trials [11].

**Table 1:** Eastern Cooperative Oncology Group (ECOG) Performance Status Scale Source: Ref. [11] (Oken MM, Creech RH, Tormey DC, Horton J, Davis TE, McFadden ET, Carbone PP. Toxicity and response criteria of the Eastern Cooperative Oncology Group. Am J Clin Oncol. 1982 Dec;5(6):649-655. PMID: 7165009) Credit: The ECOG Performance Status Scale was developed by the Eastern Cooperative Oncology Group (ECOG), now the ECOG-ACRIN Cancer Research Group, and published in 1982. To learn more, visit ecog-acrin.org/scale.

Score	Definition
0	Able to perform activities without any problems. Can carry out daily life without restrictions, just like before the disease onset.
1	Physically demanding activities are restricted; however, walking is possible, and light work or seated tasks can be performed.
2	Able to walk and perform all personal care independently; however, unable to work. Spends more than 50% of the daytime out of bed.
3	Able to perform only limited personal care. Spends more than 50% of the daytime in bed or in a chair.
4	Completely immobile. Unable to perform any personal care. Spends the entire day in bed or in a chair.
5	Dead.

Frailty

The Cardiovascular Health Study (CHS) criteria, introduced by Fried et al., are the most widely recognized standards for diagnosing frailty both globally and in Japan [12]. This phenotypic model summarizes signs expressed due to age-related functional decline for frailty screening. Experts in geriatric medicine developed the J-CHS criteria as the Japanese version of the CHS criteria [13]. The J-CHS criteria define frailty based on five key indicators: (1) unintentional weight loss, (2) persistent exhaustion, (3) decreased physical activity, (4) slow gait speed, and (5) diminished muscle strength. Individuals exhibiting three or more of these factors are classified as frail, while those with one or two are considered pre-frail. Conversely, individuals without any of these characteristics are regarded as robust [12,13]. Grip strength, used to assess frailty-related muscle weakness, was measured using the TOEI LIGHT hand dynamometer (Takei Scientific Instruments Co., Ltd., Niigata, Japan) with the dominant hand.

Activities of daily living evaluation

Activities of daily living (ADLs) were evaluated using the functional independence measure (FIM) and the Barthel Index (BI). The FIM comprises 18 items: 13 motor-related and five cognitive-related tasks. Each item is scored on a seven-point scale, with the highest possible score being 126 [14]. The BI evaluates 10 aspects of ADLs, assigning each a score ranging from five to 15 points, with a maximum achievable score of 100 [15].

Adverse events

All adverse events were defined and classified according to severity using the Common Terminology Criteria for Adverse Events (CTCAE v4.0). The adverse events listed in the CTCAE are categorized into 26 organ system classes, ranging from allergic reactions to various organ dysfunctions, with further subcategories provided under each class [16]. Adverse events were categorized into five levels according to their severity, from Grade 1 ("mild") to Grade 5 ("fatal outcome due to an adverse event"), while unclassified cases were labeled as "no Grade" (Table 2). Additionally, there were cases in which multiple adverse events of varying severity occurred in the same patient. Medical records created by physicians during the hospitalization period were used for data collection.

**Table 2:** Severity of adverse events *Preparation of meals, shopping for daily necessities and clothing, using the telephone, and managing finances; **Able to bathe, dress and undress, eat meals, use the toilet, and take medications independently, and not bedridden Source: [16]

Score	Definition
Grade 1	Mild; asymptomatic or mild symptoms; clinical or diagnostic observations only; intervention not indicated.
Grade 2	Moderate: minimal, local or noninvasive intervention indicated; limiting age-appropriate instrumental ADL*.
Grade 3	Severe or medically significant but not immediately life-threatening; hospitalization or prolongation of hospitalization indicated; disabling; limiting self-care ADL**.
Grade 4	Life-threatening consequences; urgent intervention indicated.
Grade 5	Death related to AE.

Blood data

In routine clinical practice, we considered C-reactive protein (CRP), white blood cells, neutrophils, monocytes, hemoglobin, albumin, and platelets as readily accessible parameters.

Data on blood samples obtained at the time of hospitalization or before the initiation of chemotherapy were obtained from patients’ medical records.

Ethical considerations

This research was conducted per the principles outlined in the Declaration of Helsinki and was approved by the Ethics Committee of Rakuwakai Otowa Hospital and the Research Ethics Committee of Kyoto Tachibana University (Approval Numbers: 23-00017, 24-55). All participants provided written informed consent before participating in the study.

Statistical analysis

Descriptive statistics and multiple regression analysis were used to examine the physical characteristics of male patients with lung cancer undergoing chemotherapy and their impact on length of hospital stay. After confirming a normal distribution, descriptive statistics were used to summarize the characteristics of the participants. In both univariate and multivariate analyses (multiple regression analysis), length of hospital stay was set as the dependent variable. Independent variables included age, frailty (as measured by the J-CHS), albumin levels, and CRP levels, which have been reported in previous studies on other diseases to be associated with length of hospital stay. Both forced entry and stepwise methods were used for analysis [6,8,17]. Frailty, pre-frailty, and robustness assessed using the J-CHS were converted into dummy variables and included in the analysis. A significance level of 5% was set. Statistical analysis was performed using IBM Statistical Product and Service Solutions (SPSS, version 27.0; IBM SPSS Statistics for Windows, Armonk, NY).

## Results

Study population and patient characteristics

**Figure 1:**
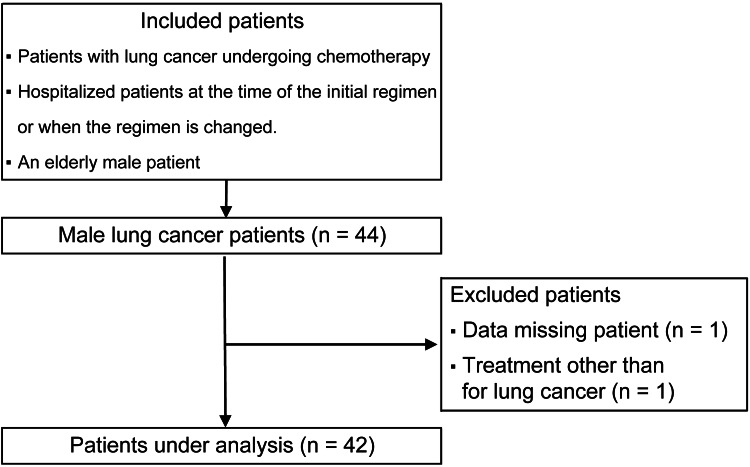
Flowchart of research participants

Overall, 44 patients were enrolled in the study. However, two patients were excluded due to incomplete data regarding length of hospital stay and the need for treatment for conditions unrelated to lung cancer (Figure 1).

The final 42 participants had average age, BMI, CCI, and PS of 70 ± 6 years, 22 ± 3, 4 ± 2 points, and 1 ± 0, respectively (Table 3). Based on the measured indicators according to the J-CHS, 16 (38.1%), 15 (35.7%), and 11 (26.2%) patients were classified as frail, pre-frail, and robust, respectively. The mean CRP and albumin levels were 1.7 ± 2.1 mg/dL and 3.7 ± 0.5 g/dL, respectively (Table 4). A total of 76 chemotherapy-related adverse events occurred during hospitalization. Based on severity, Grade 1 adverse events were the most common, accounting for 38 cases (50%). The most common individual adverse event was rash, with eight cases (10.5%) (Table 5).

**Table 3:** Characteristics of research participants Mean±SD; BMI, body mass index; CCI, Charlson comorbidity index; PS, performance status

Characteristic	n=42
Age in years	70±6
Height (cm)	167±6
Weight (kg)	62±10
BMI (kg/m^2^)	22±3
CCI (point)	4±2
PS	1±0
Need for care (yes/no)	10/32
Cohabitation (yes/no)	29/13
Staging classification (n, %)	
ⅰB	1 (2.4)
ⅱA	2 (4.8)
ⅱB	6 (14.3)
ⅲA	1 (2.4)
ⅲB	2 (4.8)
ⅳA	9 (21.4)
ⅳB	17 (40.5)
Unknown	4 (9.5)
Types of cancer (n, %)	
Small cell lung cancer	8 (19)
Adenocarcinoma	22 (52.4)
Squamous cell carcinoma	3 (7.1)
Others	9 (21.4)
Regimen (n, %)	
Cytotoxic chemotherapy drugs	40 (95.2)
Targeted molecular drugs	2 (4.8)

**Table 4:** Measurement results Mean±SD; FIM, Functional independence measure; BI, Barthel index; CRP, C-reactive protein; WBC, White blood cell count; Alb, Albumin; Neutro, Neutrophils; Mo, Monocytes; Hb, Hemoglobin; PLT, Platelet

Parameter	n = 42
At the time of admission	
Frail (n,%)	16 (38.1)
Pre-frail (n,%)	15 (35.7)
Robust (n,%)	11 (26.2)
Grip strength (㎏)	29±7
Walking speed (m/sec)	1.2±0.3
Initial FIM (score)	121±8
Initial BI (score)	96±8
CRP (mg/dL)	1.7±2.1
WBC (×10^3^/μL)	6.5±2.9
Alb (g/dL)	3.7±0.5
Neutro (/μL)	4630±2593
Mo (％)	9.5±4.4
Hb (g/dL)	12.4±2.3
PLT (×10^3^/μL)	262±129
At the time of discharge	
Discharge FIM (score)	123±7
Discharge BI (score)	98±6
Length of stay (days)	17±8

**Table 5:** Types, severity, and frequency of adverse events

Adverse events (n,%)	Grade 1	Grade 2	Grade 3	Grade 4	Grade 5	No Grade
Total	38 (50)	16 (21.1)	11 (14.5)	9 (11.8)	0 (0)	2 (2.6)
Joint pain	1 (1.3)	1 (1.3)	-	-	-	-
Phlebitis	1 (1.3)	-	-	-	-	-
Hypertension	-	-	-	-	-	1 (1.3)
Headache	-	1 (1.3)	-	-	-	-
Numbness	1 (1.3)	-	-	-	-	-
Rash	8 (10.5)	-	1 (1.3)	-	-	-
Stomatitis	3 (3.9)	-	-	-	-	-
Hair loss	2 (4.8)	-	-	-	-	-
Hiccups	2 (4.8)	-	-	-	-	-
Loss of appetite	3 (3.9)	2 (4.8)	-	-	-	-
Fatigue	6 (7.9)	2 (4.8)	-	-	-	-
Constipation	2 (4.8)	1 (1.3)	-	-	-	-
Diarrhea	1 (1.3)	-	1 (1.3)	-	-	-
Fever	2 (4.8)	1 (1.3)	-	-	-	-
Febrile neutropenia	-	-	-	-	-	4 (5.3)
Anemia	-	-	1 (1.3)	-	-	-
Leukopenia	-	2 (4.8)	2 (4.8)	2 (4.8)	-	-
Neutropenia	-	4 (5.3)	2 (4.8)	7 (9.2)	-	-
Thrombocytopenia	3 (3.9)	-	4 (5.3)	-	-	-
Elevated liver enzymes	3 (3.9)	2 (4.8)	-	-	-	-

Multivariate analyses

A multiple regression analysis was performed using length of hospital stay as the outcome variable. An observation of the correlation matrix revealed that no variables had an r > 0.8. Therefore, all variables were included in the analysis. All variance inflation factor values were <10, indicating the absence of multicollinearity. The univariate analysis showed a significant association between CRP (t = 2.354 (95% CI: 0.191-2.515), p = 0.024) and albumin (t = -3.624 (95% CI: -14.025 to -3.967), p < 0.001) (Table 6). Multivariate analysis revealed that the albumin level at admission (t = -0.512 (95% CI: -14.025 to -3.967), p < 0.001) was associated with length of hospital stay (Table 7).

**Table 6:** Results of univariate analysis CCI, Charlson comorbidity index; PS, performance status; FIM, functional independence measure; BI, Barthel index; CRP, C-reactive protein; Alb, Albumin

	Unstandardized coefficient		Standardized coefficient			
Variable	B	SE	β	t-value	p-value	95％ CI
Age	-0.21	0.208	-0.157	-1.008	0.32	-0.631–0.211
CCI	-0.449	0.674	-0.105	-0.665	0.51	-1.812–0.914
PS	4.698	2.834	0.257	1.658	0.105	-1.034–10.43
Frailty	-2.917	1.569	-0.282	-1.859	0.07	-6.089–0.255
Initial FIM	-0.194	0.158	-0.191	-1.231	0.225	-0.513–0.124
Initial BI	-0.259	0.151	-0.263	-1.723	0.093	-0.564–0.045
CRP	1.353	0.575	0.349	2.354	0.024	0.191–2.515
Alb	-8.996	2.482	-0.512	-3.624	<0.001	-14.025–3.967

**Table 7:** Results of multivariate analysis Alb, Albumin

	Unstandardized coefficient		Standardized coefficient			
Variable	B	SE	β	t-value	p-value	95% CI
Constant	50.198	9.285		5.406	<0.001	31.385–69.012
Alb	-8.996	2.482	-0.512	-3.624	<0.001	-14.025–-3.967

Analysis of variance revealed significant results, with an adjusted R² of 0.262. An adjusted R² value of approximately 0.26 indicates a mild level of model fit, commonly interpreted as limited or weak explanatory power in the social and medical sciences [18,19]. The Durbin-Watson statistic was 2.111, and no outliers with predicted values exceeding ±3 SD from the observed values were detected. Levene's test showed a significant difference in variances between the groups (F = 3.321; p = 0.005). The Shapiro-Wilk test indicated that albumin levels during the hospitalization period (p = 0.560) met the normality assumption.

## Discussion

This study focused on older male patients with lung cancer undergoing their course of chemotherapy during standard hospitalization, aiming to identify factors influencing length of hospital stay. Our results revealed that albumin levels are a prognostic factor for length of hospital stay in male patients with lung cancer undergoing chemotherapy, despite the occurrence of many adverse events. This study is the first to thoroughly evaluate the prognostic value of inflammatory markers in male patients with lung cancer. Furthermore, our findings suggest that albumin levels may be related to length of hospital stay, independently of comorbidities or physical function status at the time of admission.

Chemotherapy and hospitalization period

The average hospitalization period for patients with lung cancer who have undergone chemotherapy is reportedly 18 days [4]. According to the Organization for Economic Cooperation and Development data, the average length of hospital stay for acute care in Japan is reportedly 16 days [20]. However, in this study, the average was 17 days for patients with lung cancer undergoing chemotherapy.

Albumin and the hospitalization period

Albumin is a parameter that is often associated with cancer cachexia. Previous studies have reported that pre-treatment albumin levels have prognostic significance [21-23]. However, most studies assess albumin in combination with other clinical laboratory parameters, such as the albumin-to-alkaline phosphatase ratio, albumin-to-fibrinogen ratio, or albumin/globulin ratio. In this study, considering the ease of recording these parameters in routine clinical practice, we evaluated the significance of albumin alone and confirmed that it is a negative prognostic factor. Therefore, albumin is proposed as a useful clinical laboratory parameter for predicting length of hospital stay.

Recently, a negative correlation was observed between albumin and CRP levels in older adults. Therefore, albumin is considered an inflammatory marker, potentially serving as a screening tool for inflammation, guiding therapeutic interventions, and avoiding excessive treatment of inflammatory patients [24].

Albumin, a key indicator of inflammation, is an important predictor of disease severity in patients, particularly those with chronic or critical illnesses. Inflammatory cytokines associated with cancer, including interleukin-6 (IL-6), interleukin-1, and tumor necrosis factor-alpha (TNF-α), suppress albumin synthesis and contribute to the development of cancer cachexia [25]. Albumin is considered a separate prognostic factor for various cancer types [26].

CRP, a protein produced by hepatocytes, is classified as an acute-phase protein. Its production is regulated by cytokines such as IL-6 and TNF-α [27]. CRP has been linked to tumor malignancy and physical cachexia. Numerous studies have reported that cancer patients with elevated CRP levels tend to have poorer prognoses compared to those with normal CRP levels [28]. In this study, univariate analysis demonstrated a correlation between length of hospital stay and both CRP and albumin levels. However, multivariate analysis revealed that albumin, rather than CRP, was more strongly associated with length of hospital stay in older male patients with lung cancer undergoing chemotherapy.

Albumin and CRP levels in older individuals also serve as inflammatory markers [24]. Furthermore, CRP is an acute-phase protein influenced by IL-6 and TNF-α, linked to tumor aggressiveness and cachexia. Studies have shown that patients with cancer with elevated CRP levels tend to have a worse prognosis [26-28].

Studies investigating the association between albumin and length of hospital stay across various diseases have reported that low albumin levels at admission correlate with prolonged hospitalization [9]. From this, it is inferred that low albumin level at admission is associated with prolonged hospitalization in male patients with lung cancer as well.

Limitations

The study's limitations include its single-center design and exclusion of social factors beyond cohabitation status and the presence or absence of care needs. The small sample size may have led to decreased statistical power. Not all patients with lung cancer were included, raising the possibility of selection bias during patient recruitment. Future research should explore the generalizability of the results, including conducting multicenter collaborative studies and examining social factors such as social environment, roles, and economic conditions.

Moreover, due to the limited sample size, we were unable to include disease-specific factors, such as cancer stage, chemotherapy regimen, and cancer type, in the analysis. Consequently, their influence on length of hospital stay could not be evaluated.

## Conclusions

This study demonstrated that albumin levels during hospital admission serve as an independent predictor of prolonged hospitalization in older male patients with lung cancer undergoing chemotherapy. Albumin is a marker that reflects inflammatory status, and its early assessment may be useful for optimizing patient management and discharge planning. Future studies should investigate sociodemographic factors in multicenter settings.
